# Human leukocyte antigen class 1 genotype distribution and analysis in persons with active tuberculosis and household contacts from Central Uganda

**DOI:** 10.1186/s12879-016-1833-3

**Published:** 2016-09-23

**Authors:** Helen K. Buteme, Rebecca Axelsson-Robertson, Lina Benson, Moses L. Joloba, W. Henry Boom, Gunilla Kallenius, Markus Maeurer

**Affiliations:** 1 Department of Medical Microbiology, School of Biomedical Sciences, College of Health Sciences, Makerere University, P.O Box 7072, Kampala, Uganda; 2 Department of Clinical Science and Education, Karolinska Institute, Södersjukhuset, SE-118 83 Stockholm, Sweden; 3 Department of Microbiology, Tumor and Cell Biology (MTC), Nobels vag 16, KI Solna Campus Karolinska Institute, Box 280, SE-171 77 Stockholm, Sweden; 4 Tuberculosis Research Unit, Case Western Reserve University and University Hospitals’ Case Medical Center, 10900 Euclid Avenue, BRB 1031, Cleveland, OH 44106-4984 USA

**Keywords:** HLA, Genotype, Tuberculosis, Uganda

## Abstract

**Background:**

To determine the distribution of Human leukocyte antigen (HLA) class I genotypes in a Ugandan population of persons with tuberculosis (TB) and establish the relationship between class I HLA types and *Mycobacterium tuberculosis* (MTB) disease.

**Methods:**

Blood samples were drawn from HIV negative individuals with active TB and HIV negative household controls. DNA was extracted from blood samples and HLA typed by the polymerase chain reaction-sequence specific primer method. The allelic frequencies were determined by direct count.

**Results:**

HLA-A*02, B*15, C*07, C*03, B*58, C*04, A*01, A*74, C*02 and A*30 were the dominant genotypes in this Ugandan cohort. There were differences in the distribution of HLA types between the individuals with active TB and the household controls with only HLA-A*03 allele showing a statistically significant difference (*p* = 0.017 crude; OR = 6.29 and *p* = 0.016; OR = 11.67 after adjustment for age). However, after applying the Benjamini and Hochberg adjustment for multiple comparisons the difference was no longer statistically significant (*p* = 0.374 and *p* = 0.176 respectively).

**Conclusions:**

We identified a number of HLA class I alleles in a population from Central Uganda which will enable us to carry out a functional characterization of CD8+ T-cell mediated immune responses to MTB. Our results do not show a positive association between the HLA class I alleles and TB in this Ugandan population however the study sample was too small to draw any firm conclusions about the role of HLA class I alleles and TB development in Uganda.

**Electronic supplementary material:**

The online version of this article (doi:10.1186/s12879-016-1833-3) contains supplementary material, which is available to authorized users.

## Background

Uganda is among the world’s 22 countries with a high TB burden [1]. In addition to the high prevalence the case-finding rate is low and approximately 58 % of Uganda’s TB cases remain undetected [2]. One of the reasons for the difficulty in detecting the majority of TB cases is due to the poor performance characteristics of diagnostic methods such as sputum smear microscopy, chest x-rays and tuberculin skin test (TST), that are available in Uganda [3–5]. Culturing for tubercle bacilli, although accurate, takes too long. Other rapid and reliable tests such as polymerase chain reaction (PCR) and enzyme-linked immunospot assay (ELISPOT) are unavailable for diagnosis in Uganda, due to their high costs, their need for expensive equipment and skilled manpower [3, 6].

Currently, *Mycobacterium bovis* bacillus Calmette-Guerin (BCG) is the only TB vaccine available for humans, however, despite its widespread use, it is not effective in providing protection against pulmonary TB (PTB) in developing countries [7–9]. Human leukocyte antigens (HLA) are important molecules for the initiation of adaptive immune responses to infectious agents. HLA diversity plays a crucial role in the host-pathogen interaction and can affect the rates of disease acquisition and outcome [10–12]. An individual’s HLA profile can determine resistance or susceptibility to certain infectious diseases, e.g., Vijaya et al. [13] showed Indian patients with the HLA-B51 allele were predisposed to PTB while the HLA-B52 allele conferred protection from PTB on individuals carrying it [13]. In studies by Khomenko et al. [14], the association between TB and HLA-A, HLA-B and HLA-C varied between different populations in the Soviet Union although ethnically related populations showed associations with the same HLA allele [14]. Despite the fact that little significant association between HLA class I alleles and MTB susceptibility has been found, interallelic variations in the HLA class I locus may still have an impact on immune recognition due to their ability to present different peptide repertoires and thereby influencing immune recognition by antigen-specific T-cells. The identification of HLA alleles in individuals exposed to TB in Uganda as well as determining which alleles are associated with the various TB outcomes will help in screening individuals in high-risk areas in Uganda for susceptibility to TB and also to predict their resistance to MTB infection or their progression to active TB. This would inevitably lead to better clinical management of TB. In addition, the identification of the common alleles in the Ugandan TB population will be useful in the development and evaluation of TB vaccines that will effectively work on a broader range of the high risk individuals in a Ugandan population. The aim of this study was to determine the distribution of HLA class I genotypes in a Ugandan population of persons with TB and to establish the relationship between class I HLA types and *Mycobacterium tuberculosis* (MTB) disease.

## Methods

### Subjects

Blood samples were obtained from HIV negative Ugandan adults (18 years or older) from Kawempe division, one of the five administrative divisions in Kampala (Central Uganda), who presented with PTB (confirmed with subsequent positive culture) and were the first TB case identified in the household (index case). Blood samples were also obtained from HIV negative individuals of any age who had resided in the household for at least seven consecutive days during the 3 months prior to the diagnosis of TB in the index case and who were TST positive (household contacts). TST induration ≥10 mm was considered indicative of latent TB infection.

### HLA typing at the HLA-A, HLA-B and HLA-C loci

DNA was extracted from 200ul of the whole blood samples using an Epicentre MasterPure DNA purification kit. The quantity and purity of the DNA was determined by electrophoresis on a 2.5 % agarose gel stained with ethidium bromide. The DNA was then genotyped for HLA class I at the loci A, B and C using OneLambda HLA class I locus A, B and C polymerase chain reaction-sequence specific primer (PCR-SSP) typing kit following the manufacturer’s instructions. The PCR products were analysed by electrophoresis on a 5 % agarose gel stained with ethidium bromide. Images of the gels were taken using a UV transilluminator and the HLA types of the samples were determined by the presence of bands in appropriate wells. The HLA alleles at the 2-digit level were assigned according to the patterns specified by the manufacturer.

### Statistics

Baseline characteristics were tested for distributional differences between the active TB subjects and the household contacts using Fisher’s exact test (categorical variables) and *t*-test (continuous variables). The HLA alleles were counted directly and the allelic frequencies determined using the formula alleles/(2 x N). Differences in allelic frequency between groups were presented with Odds Ratio (OR), 95 % confidence interval (CI) and tested with Fisher’s exact test. Since age differed between the two groups the results were recalculated adjusting for age by means of logistic regression. Adjustment for multiple testing was performed using the Benjamini-Hochberg (FDR) method, taking into account the number of tests performed within each HLA type. All statistical analyses were performed in R version 3.1.3 (R Foundation for Statistical Computing). The level of significance was 5 % and all reported p-values and CI are 2-sided.

## Results and discussion

### Subjects

Seventy-seven subjects were included in the study of which 32 were confirmed TB cases (index cases) and 45 being TST positive household contacts. Fifty-four and a half percent of the subjects were female with 50 % of the index cases and 57.8 % of the household contacts being female. There was no statistically significant difference in the percentage of male and female subjects between the two groups (*p* = 0.643) (Additional file 1: Table S1). The mean age of all the subjects was 22.7 years with a mean of 30.8 years (range 18 to 54 years) for index cases and a mean of 16.9 years (range 1 to 44) for household contacts. There was a statistically significant difference in age between the two groups (*p* < 0.001) (Additional file 1: Table S1) therefore age might be considered a confounding factor in this study. However, previous studies have shown that after infection children, particularly those under 2 years old, are at the highest risk of progression to active disease [15, 16] with the lowest risk in children aged between 5 - 10 years [15, 17]. Adults aged 65 years and above are at an increased risk of infection and reactivation of latent TB [18]. Children older than 10 years showed similar disease outcomes to those of adults [16].

The population studied resided in Kawempe division in Kampala district, Central Uganda. Kampala is a melting pot of tribes from various regions in Uganda that are categorized into 5 main ethnicities: Eastern Lacustrine Bantu, Western Lacustrine Bantu, Eastern Nilotic, Western Nilotic and Central Sudanic. Patient samples included subjects who self-reported that they belonged to particular tribes which were then grouped into the following 4 ethnicities: Eastern Lacustrine Bantu (74 %), Western Lacustrine Bantu (18.2 %), Eastern Nilotic (3.9 %) and Western Nilotic (3.9 %). None of the subjects reported being Central Sudanic. The majority of the subjects are Eastern Lacustrine Bantu who are mainly found in Central and Eastern Uganda. There were no statistically significant differences between index cases and household contacts in terms of tribe (*p* = 0.984) and ethnicity (*p* = 0.882) (Additional file 1: Table S1).

Thus the family based controls used in this study are appropriate in an urban ethnically heterogenous population to reduce confounding factors such as race, ethnicity and genetic background as well as socioeconomic factors [19, 20].

### Allelic frequencies

There were 16 distinct HLA-A alleles, 24 distinct HLA-B alleles and 10 distinct HLA-C alleles at the 2-digit representation among the 77 subjects. There were a few cases of ambiguity that arose with some individuals (*n* = 39) having more than 2 alternatives to an allele at a locus (A*02/A*68, A*11/A*15, A*11/A*23, A*23/A*33, A*26/A*A23, B*41/B*45, B*51/B*53, C*02/C*03/C*15, C*03/C*04, C*06/C*07, C*06/C*12 and C*08/C*12). This ambiguity was possibly due to methodological limitations, i.e., similarity in reactivity patterns of the alleles meaning that the PCR-SSP was unable to distinguish them. (Additional file 2: Table S2) shows the allelic frequencies of the HLA-A, HLA-B and HLA-A C loci of the population. HLA-A*02 (23 %), A*01 and A*74 (both 10 %) were found to be the most common HLA-A alleles, HLA-B*15 (19 %), B*58 (15 %) and B*42 and B*44 (both 8 %) were found to be the most common HLA-B alleles and HLA-C*07 (19 %), C*03 (18 %) and C*04 (15 %) were found to be the most common HLA-C alleles in the study cohort.

Our study identified approximately the same number of alleles as previous studies conducted on Ugandans from Kampala by Kijak et al. and Cao et al. but did not identify several HLA alleles that were identified in Kijak and/or Cao’s studies (A*25, A*31, A*69, A*31, B*37, B*38, B*39, B*52, B*55, B*56, B*73, B*82, C*01, C*05 and C*18) [21, 22]. This may be attributed to the small sample size in our study as well as due to the different genetic backgrounds for the tested study populations. However there was a large overlap in the different studies for the most frequent HLA class I allele: both our group and the previous mentioned groups identified HLA-A*02 to be the most common HLA-A allele present while HLA-B*15 and B*58 were the most prevalent HLA-B alleles present. HLA-C also showed a large overlap between the three different studies with HLA-C*07 and C*04 always being among the top three most common alleles. Our study identified the most common HLA class I alleles in this particular Ugandan population and this information is important as the HLA combinations vary from individual to individual meaning that each person has a different response to TB infection.

### HLA association

The allelic frequencies differed numerically between the active TB patients and the household contacts. In the active group the following HLA alleles were the most common: HLA-A*02 (20.31 %), A*03 (12.50 %), A*30 (10.94 %), A*74 (10.94), HLA-B*15 (23.44 %), B*58 (14.06 %) and B*42 (7.81 %) and HLA-C*03 (20.31 %), C*07 (18.75 %) and C*04 (10.94 %). The following HLA alleles were most common in the household contacts: HLA-A*02 (24.44 %), A*01 (12.22 %) and A*74 (10 %), HLA-B*58 (15.56 %), B*15 (15.56 %) and B*44 (8.89 %) and C*07 (18.89 %), C*04 (17.78 %), and C*03 (15.56 %). (Additional file 3: Table S3) shows the allelic frequencies of the 2 groups and the p values. There was no statistically significant difference concerning the expression of different HLA alleles between the two groups except for the HLA-A*03 allele (12.50 % versus 2.22 %) (*p* = 0.017; OR = 6.29, 95 % CI 1.29-30.68) however after Benjamini-Hochberg (FDR) adjustment *p* = 0.374. After adjusting for age between the two groups for the HLA-A*03 allele *p* = 0.016; OR = 11.67, 95 % CI 1.58-86.26 and after Benjamini-Hochberg (FDR) adjustment *p* = 0.176. Despite the loss of statistical significance, 8out of 32 active TB patients had the HLA-A*03 allele but only two of the household contacts out of 45 had the HLA-A*03 allele - this allele needs to be looked further in a study with a larger sample size before drawing any firm conclusions. A study carried out in 1990 showed that A*03 had a positive association with TB in the Kazakh although it had no significance in the other Soviet groups or was not present in their HLA genotype [14]. This same allele had no positive association in Iraqi and Indian PTB patients [23, 24].

## Conclusions

Uganda has one of the highest TB incidences in the world. A prophylactic vaccine would be more useful than TB medication in combating the disease. Despite the small size of the study we have been able to identify a number of HLA class alleles in a population from Central Uganda and this will enable us to carry out the functional characterization of CD8 immune responses to MTB antigens and this will identify the most effective MTB antigens in producing protective immune responses to MTB infection. This information would be important in the design of a vaccine for TB.

Our results do not show a positive association between any of the HLA class I alleles identified and TB. However, a larger study investigating the association between the HLA-A*03 allele and TB and also trying to identify other alleles with positive associations to TB would be beneficial as identifying such high risk individuals and administering the appropriate interventions would go a long way towards improving the clinical management of TB and reducing the spread of TB in the population.

## Additional files

Additional file 1:
**Table S1.** Distributional differences in baseline characteristics between active TB patients and household contacts in the study. Table comparing the baseline characteristics of active TB patients and household contacts in the study. (XLS 26 kb) Additional file 2:
**Table S2.** Total allelic frequencies of the study subjects from Kawempe, Kampala. Table of the total allele frequencies for HLA-A, HLA-B and HLA-C alleles of both active TB patients and household contacts in the study. (XLS 28 kb) Additional file 3:
**Table S3.** Comparison of allelic frequencies between active TB patients and household contacts in the study. Table comparing the allele frequencies for HLA-A, HLA-B and HLA-C alleles between active TB patients and household contacts in the study. (XLS 49 kb)

## Abbreviations

HLA: Human leukocyte antigen
TB: Tuberculosis
MTB: *Mycobacterium tuberculosis*
HIV: Human immunodeficiency virus
DNA: Deoxyribonucleic acid
PCR-SSP: Polymerase chain reaction-sequence specific primer
TST: Tuberculin skin test
PCR: Polymerase chain reaction
ELISPOT: Enzyme-linked immunospot assay
BCG: Bacillus Calmette-Guerin
PTB: Pulmonary TB
CWRU: Case Western Reserve University
JCRC: Joint clinical research center
UNCST: Uganda National Council for Science and Technology
